# Myeloperoxidase inhibition in mice alters atherosclerotic lesion composition

**DOI:** 10.1371/journal.pone.0214150

**Published:** 2019-03-19

**Authors:** Rachel J. Roth Flach, Chunyan Su, Eliza Bollinger, Christian Cortes, Andrew W. Robertson, Alan C. Opsahl, Timothy M. Coskran, Kevin P. Maresca, Edmund J. Keliher, Phillip D. Yates, Albert M. Kim, Amit S. Kalgutkar, Leonard Buckbinder

**Affiliations:** 1 Internal Medicine Research Unit, Pfizer Inc., Cambridge, Massachusetts, United States of America; 2 Drug Safety Research and Development Global Pathology, Pfizer Inc., Groton, Connecticut, United States of America; 3 Early Clinical Development, Pfizer Inc., Cambridge, Massachusetts, United States of America; 4 Medicine Design, Pfizer Inc., Cambridge, Massachusetts, United States of America; Max Delbruck Centrum fur Molekulare Medizin Berlin Buch, GERMANY

## Abstract

Myeloperoxidase (MPO) is a highly abundant protein within the neutrophil that is associated with lipoprotein oxidation, and increased plasma MPO levels are correlated with poor prognosis after myocardial infarct. Thus, MPO inhibitors have been developed for the treatment of heart failure and acute coronary syndrome in humans. 2-(6-(5-Chloro-2-methoxyphenyl)-4-oxo-2-thioxo-3,4-dihydropyrimidin-1(2*H*)-yl)acetamide PF-06282999 is a recently described selective small molecule mechanism-based inactivator of MPO. Here, utilizing PF-06282999, we investigated the role of MPO to regulate atherosclerotic lesion formation and composition in the *Ldlr*^-/-^ mouse model of atherosclerosis. Though MPO inhibition did not affect lesion area in *Ldlr*^-/-^ mice fed a Western diet, reduced necrotic core area was observed in aortic root sections after MPO inhibitor treatment. MPO inhibition did not alter macrophage content in and leukocyte homing to atherosclerotic plaques. To assess non-invasive monitoring of plaque inflammation, [^18^F]-Fluoro-deoxy-glucose (FDG) was administered to *Ldlr*^-/-^ mice with established atherosclerosis that had been treated with clinically relevant doses of PF-06282999, and reduced FDG signal was observed in animals treated with a dose of PF-06282999 that corresponded with reduced necrotic core area. These data suggest that MPO inhibition does not alter atherosclerotic plaque area or leukocyte homing, but rather alters the inflammatory tone of atherosclerotic lesions; thus, MPO inhibition could have utility to promote atherosclerotic lesion stabilization and prevent atherosclerotic plaque rupture.

## Introduction

Atherosclerosis is a chronic inflammatory disease that is hallmarked by hyperlipidemia-induced vascular inflammation and inflammatory cell recruitment to the vessel wall [1–4]. Though statins are a mainstay of treatment to reduce LDL and therefore prevent atherosclerotic lesions and subsequent cardiac pathologies, anti-inflammatory therapies could have further benefit beyond lipid lowering. Indeed, the recent CANTOS clinical trial for canakinumab (anti-IL-1β) demonstrated clinical benefit to prevent cardiovascular events independent of lipid lowering[5]. Atherosclerotic plaques are mainly comprised of lipid-laden macrophage-derived foam cells, and less is understood about the role of the neutrophil and other inflammatory cells in atherosclerotic lesion progression and remodeling[4]. Recent studies have suggested that atherosclerosis is initiated by early neutrophil recruitment followed by neutrophillic extracellular trap (NET) formation, but it is less clear what role the neutrophil plays in established atherosclerotic lesions[6,7]. Myeloperoxidase (MPO) is an abundantly expressed protein in azurophilic granules and to a much lesser extent in activated macrophages, and high levels of MPO are expressed within atherosclerotic plaques[8–11]. MPO is a key mediator of oxidative stress, as it catalyzes the production of hypochlorous acid (bleach) from hydrogen peroxide and chloride ion[12]. Not only does this HOCl formation damage tissue and promote further inflammatory cell recruitment[13], but it also has a well-established role to oxidize lipoproteins[10,14–17]. MPO also promotes endothelial dysfunction and impairs vessel reactivity by consuming nitric oxide[18] and is also required for NET formation[19]. These events promote a feed forward cycle to drive vascular inflammation.

Atherosclerotic progression and unstable plaque rupture are precursors to cardiac ischemia and myocardial infarct (MI)[20]. Increased plasma MPO levels are highly correlated with adverse cardiovascular events such as MI[13,21,22]. Mice lacking MPO expression display protection from MI and multiple other inflammatory diseases, yet paradoxically also display increased atherosclerotic lesion area[23,24]. Nonetheless, small molecule MPO inhibitors have been generated that display high selectivity to the MPO molecule over the closely related thyroid peroxidases[25,26]. Some of these molecules have been advanced to humans for clinical trials [27,28]. In mouse models of myocardial infarct, treatment with one such irreversible inhibitor of MPO (PF-‘1355) demonstrated prevention of MI-induced scar formation and remodeling and additionally improved cardiac function[29]. The present study utilized our recently disclosed selective MPO inactivator and clinical candidate PF-06282999, a covalent and irreversible MPO inhibitor reported in Ruggeri et al that is analogous in structure to PF-‘1355[25], to inhibit MPO activity in the low density lipoprotein receptor (Ldlr) knockout (*Ldlr*^-/-^) mouse model of atherosclerosis and assess whether MPO inhibition was able to reduce atherosclerotic plaque burden or promote atherosclerotic plaque remodeling. We observed that although chronic MPO inhibitor treatment did reduce MPO activity in both plasma and aorta, MPO inhibition with PF-06282999 did not affect lesion size. However, reduced necrotic core area was observed in histological sections of aortic roots after PF-06282999 treatment. These changes occurred in the absence of significant alterations in macrophage content within the plaque or leukocyte homing to the aorta or aortic root. Finally, ^18^F FDG signal was reduced in aorta after a therapeutic treatment regimen of PF-06282999, thus providing a method by which to monitor MPO inhibition in disease.

## Materials and methods

### Animal studies

The Pfizer Institutional Animal Care and Use Committee approved all of the animal procedures. A Pfizer-specific cohort of *Ldlr*^-/-^ mice were bred at Jackson Laboratories, USA. Starting at 8–10 weeks of age, male mice were fed Western diet (0.2% cholesterol, TD 88137, Harlan Laboratories, USA) *ad libitum* for 7–16 weeks as indicated. Plasma total cholesterol values were assessed using a Siemens clinical analyzer. Mice were euthanized by CO_2_ inhalation followed by bilateral pneumothorax. No statistical methods were utilized to predict sample size. The investigators were blinded during data collection and analysis.

### PF-06282999 administration and measurement

PF-06282999 was formulated in a 1:1 solution of 1% hydroxymethylcellulose and 1% hypromellose acetate succinate in 40 mM Tris Base, pH 10.5 and was administered by oral (PO) gavage twice-a-day (BID) at concentrations of 0.5 or 1.5 mg/mL and dosed at 10 ml/kg (5 or 15 mg/kg dose). Control animals were given 10 ml/kg vehicle only. In all studies, EDTA plasma was collected one hour after the final administration of PF-06282999, and plasma PF-06282999 concentrations were assessed via mass spectrometry.

### Aortic staining

Aortas were perfused with saline followed by 10% formalin. *En face* preparations were stained with Oil Red-O in 60% isopropanol and imaged with low magnification light microscopy with a metric ruler for scale. For fluorescent microsphere quantitation, full length aortas were cleaned and placed in a 96 well plate, and for each well, 4 quadrants and 10 Z stacks were imaged in each color as well as brightfield, and RFP was quantified among all of these images and summed using an algorithm on the In Cell high content imaging system (Columbus, Perkin Elmer, USA). RFP-positive cells were normalized to aorta area as measured by *en face* preparation. Frozen aortic root sections (10 μm) were stained with haematoxylin and eosin, Oil Red O, trichrome, or DAPI and were scanned using Eslide manager (Leica Biosystems, USA). For CD68 staining, endogenous peroxidase activity was quenched with 0.3% hydrogen peroxide followed by protein block (Rodent Block M, #RBM961, BioCare, USA) and slides were incubated with 3 μg/mL CD68 antibodies (clone FA-11, #MA5-16674, Thermo Fisher Scientific, USA) or negative isotype IgG (Purified Rat IgG2a,k #553927 BD Biosciences, USA) in TBS-T (Dako Wash Buffer 10X #S3006, Agilent, USA) for 60 mins followed by peroxidase-conjugated polymer/linking reagent for 30 minutes (BioCare Rat HRP-Polymer, 1-Step, #BRR4016L). HRP-enzyme was developed using the Dako Liquid DAB Chromagen Substrate (#K3468) followed by a tap water rinse and counterstain in Mayer’s Hematoxylin (Dako #S3309). Quantification of H&E, Masson’s trichrome, Oil Red O, CD68 and necrotic core area were performed manually using image J software (NIH, USA) on serial sections on one aortic root slice per animal. The necrotic core area was quantified from the H&E section and was defined as the area within the aortic root plaque that had an absence of cellularity. Area was defined using a scale bar as a reference, which was integrated into the image by Eslidemanager software. Investigators were blinded during image analysis.

### Leukocyte homing assay

Peritoneal exudate cells were elicited by an intraperitoneal injection of 1 mL 4% thioglycollate into 8–10-week-old male C57BL/6 mice (Jackson Laboratories). After 2 days, cells were harvested by PBS wash of the peritoneal cavity. Isolated PECs were of a mixed cellular population. Cells were pooled, strained through 70 μm mesh and labeled using 76 μl FluoSpheres Carboxylate-Modified Microspheres, 2.0 m, red fluorescent (580/605), 2% solids (Thermo Fisher) per 10^7 cells for 75 minutes at 37°C to label phagocytic cells. Labeled cells were washed with PBS and resuspended at 1.5 x 10^7 cells/mL in PBS. 2x10^6 cells were injected intravenously into *Ldlr*^-/-^ mice that had been fed Western diet and treated for 7 or 14 weeks with PF-06282999 or vehicle as detailed above.

### MPO activity assay

A high- binding, half area, black plate was coated with 50 μL mouse anti-MPO capture antibodies (1:200 dilution; Abcam 16886, UK) and incubated overnight at 4°C. The plate was then washed with PBS-T and blocked with 1% BSA/PBS for 1 hour at 4°C. 50μL plasma (diluted 1:5 in PBS) or homogenized aorta samples [20 mg/mL] in lysis buffer (1x Lysis Buffer, Cell Signaling, USA with 1x Halt phosphatase and protease inhibitor cocktail, Thermo Fisher) were aliquotted followed by incubation for 2 hours at 4°C, at which time the plate was washed with PBST. 50 μL Amplex Red Assay Buffer (50 mM NaPO_4_ pH 7.4, 140 mM NaCl, 10 mM Na_2_NO_2,_ 40 μM Amplex Red, 10 μM H_2_O_2_) was added, and the plate was shaken for 25 mins and read on the SpectraMax M2 plate reader at excitation 530 nM, emission 580 nm. Activity was calculated based on a standard curve of purified MPO protein.

### F-18 FDG tracer biodistribution study

[^18^F]-Fluoro-deoxy-glucose (^18^F-FDG) was purchased commercially from PETNET, USA. ^18^F-FDG experiments were performed in male *Ldlr*^-/-^ mice fed Western diet (TD 88137, Harlan) and male wild type C57Bl6/J fed standard rodent chow (8–10 weeks on study) supplied by Jackson, not aged matched. Animals were weighed twice a week and fasted the night before the ^18^F-FDG injection. Vehicle or PF-06282999 was administered BID via PO gavage at both 5 and 15 mg/kg dose levels for 4 weeks beginning on week 12 of Western diet. ^18^F-FDG assessments were performed on week 12 (n = 5 WT) and on week 16 (*Ldlr*^-/-^ mice vehicle n = 18; 5 mg/kg n = 10 and 15 mg/kg n = 9). All animals were consciously administered ^18^F-FDG via intravenous tail injections at 0 mins (200–300 μCi, <100 μL) in a saline solution. The animals were allowed to freely move after tracer injection prior to euthanasia. A heating pad was placed under cages 30 mins prior to injections and for the subsequent time until sacrifice. At 60 mins post tracer injection, each mouse was euthanized and had the maximum amount of blood collected followed by perfusion with 10% formalin in PBS. For all mice, the heart, lungs (both), liver (whole), kidney (both), muscle and blood were collected, weighed and measured for radioactivity content. In addition, all tails and empty syringes for each animal were measured in the gamma counter to account for radioactivity reconciliation. For all mice, the maximal length of the thoracic-abdominal aorta along with aortic arch was collected and weighed in prefilled, pre-weighed and capped 10% formalin tubes, radioactivity content was measured.

### FDG gamma counting

Radioactive samples were collected and placed in pre-weighed tubes for counting purposes on a Perkin Elmer Wallac Wizard 1470. Samples were counted in racks with empty spaces between samples to reduce count spillover effects. Three ^18^F-FDG standard dilutions of known activities (0.25–1.0 μCi) were counted to calculate an efficiency factor to convert counts to units of activity (0.215 ± 0.009 CPM/DPM), and three empty tubes were counted with each run to calculate background activity. The measured counts per minute (CPM) were corrected for background, decay corrected to injection time and converted to units of standard uptake value (g/mL). Residual activity at the injection site (tail) was subtracted from the injected dose.

### Statistical analysis

**Fig 1:**
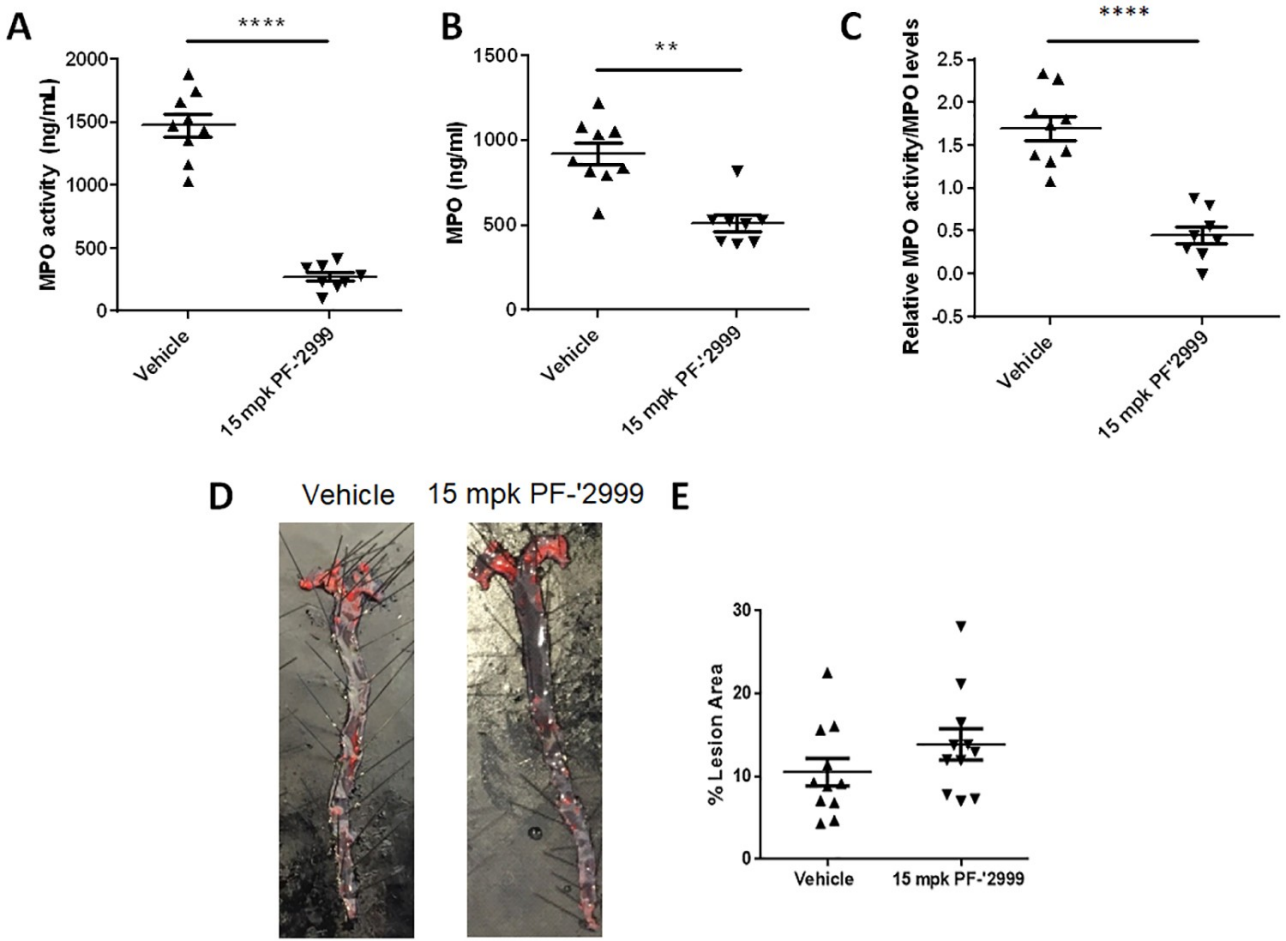
*Ldlr*^-/-^ mice treated with MPO inhibitor PF-06282999 had reduced plasma MPO activity but not atherosclerotic lesion area. *Ldlr*^-/-^ mice were fed Western diet and treated with 15 mg/kg MPO inhibitor PF-06282999 PO, BID for 14 weeks. **A-C**. MPO levels and activity were assessed in plasma one hour after the final compound dose. **A**. MPO activity, **B**. MPO levels. **C**. MPO activity normalized to MPO levels. **D-E**. Aortas were extracted, pinned en face, and stained with Oil Red O. **D**. Representative images of aortas after vehicle or PF-06282999 treatment. **E**. Quantitation of Oil Red O staining as a percentage of aorta area. (N = 8–11, **; p<0.01, ****; p<0.0001).

**Fig 2:**
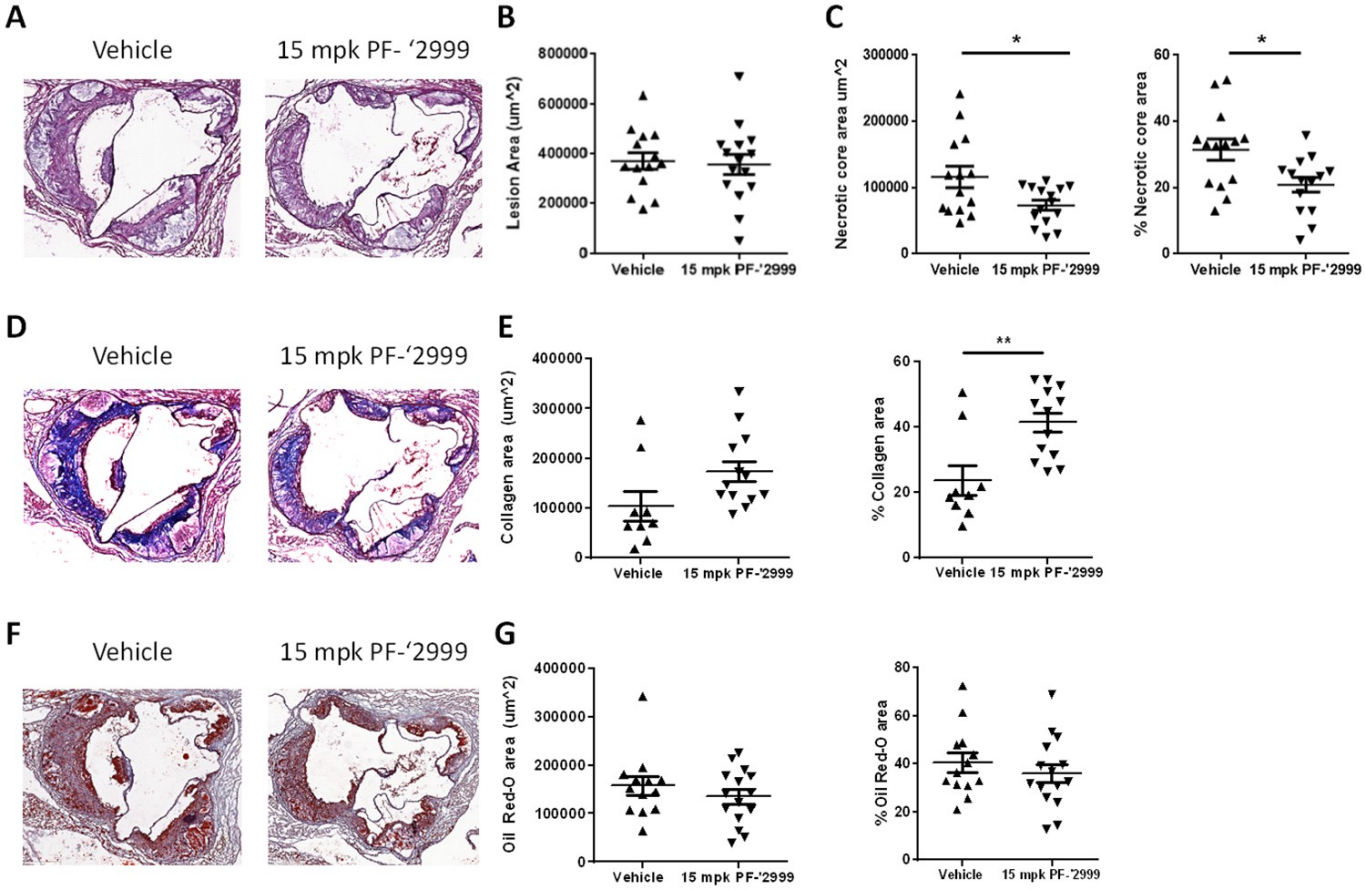
Reduced necrotic core area and enhanced percentage of collagen in aortic roots of *Ldlr*^-/-^ mice treated with MPO inhibitor PF-06282999. *Ldlr*^-/-^ mice were fed Western diet and treated with 15 mg/kg MPO inhibitor PF-06282999 PO, BID for 14 weeks. Hearts were isolated, and aortic roots were sectioned and stained with H&E (**A-C**), Masson’s Trichrome (**D-E**), or Oil Red-O (**F-G**). **A**. Representative images of H&E stained aortic roots from vehicle or PF-06282999-treated animals. **B**. Quantitation of lesion area. **C**. Left panel, quantitation of necrotic core area. Right panel, quantitation of necrotic core area as a percentage of lesion area. **D**. Representative images of Masson’s trichrome stained aortic roots from vehicle or PF-06282999-treated animals. **E**. Left panel, quantitation of collagen area. Right panel, quantitation of collagen area as a percentage of lesion area. **F**. Representative images of Oil Red O stained aortic roots from vehicle or PF-06282999-treated animals. **G**. Left panel, quantitation of Oil Red O area. Right panel, quantitation of Oil Red O area as a percentage of lesion area. (N = 9–15, *; p<0.05, **; p<0.01). Aortic root images were taken at 5x magnification.

**Fig 3:**
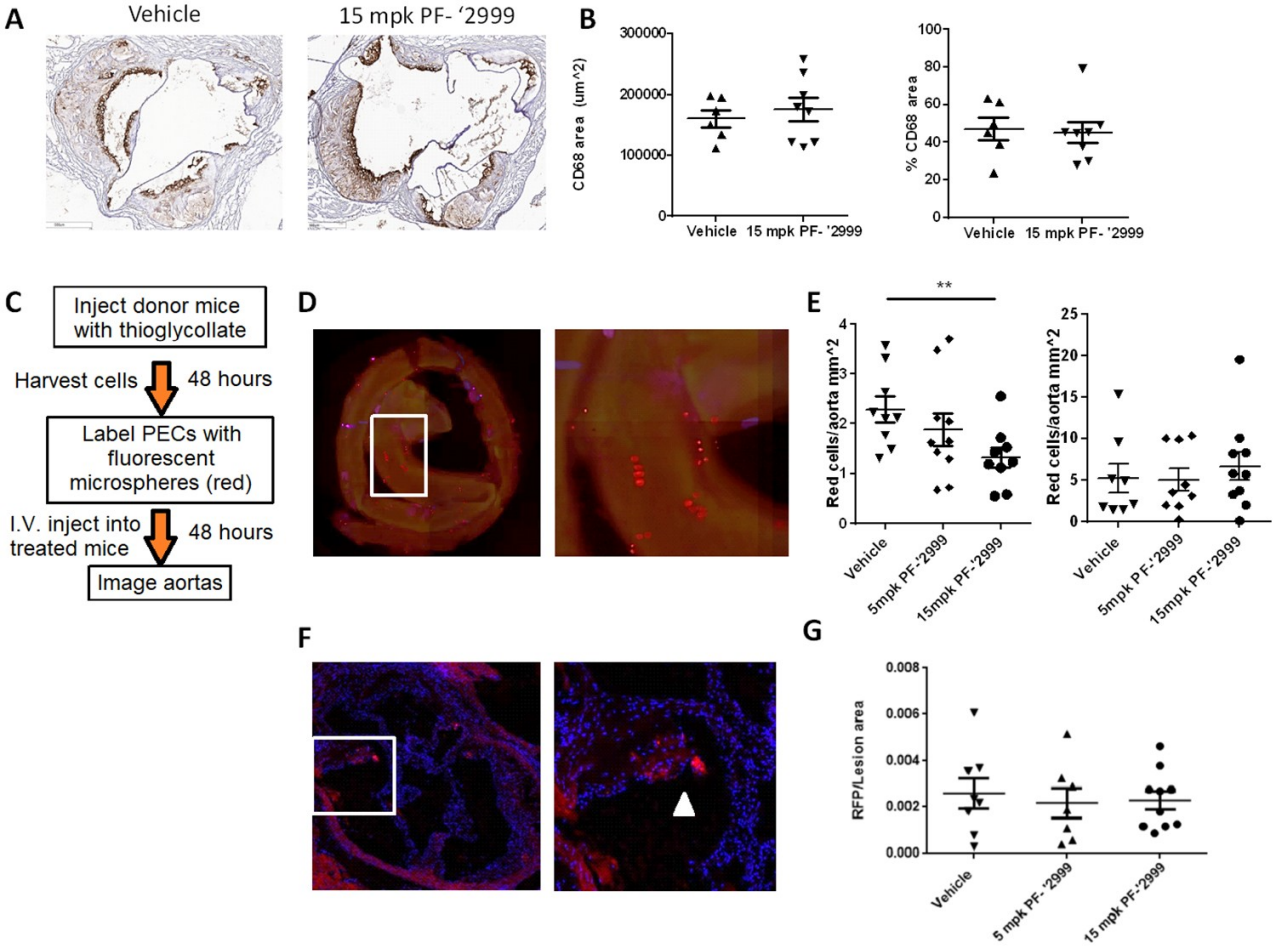
Unaltered macrophage content in and immune cell trafficking to plaques after MPO inhibition. *Ldlr*^-/-^ mice were fed Western diet and treated with 5 or 15 mg/kg MPO inhibitor PF-06282999 PO, BID for 7 or 14 weeks. **A-B**. Hearts were isolated and aortic roots were sectioned and stained with CD68 antibodies. **A**. Representative images from vehicle or PF-6282999-treated animals (images were taken at 5x magnification). **B**. Left panel, quantitation of CD68-positive area. Right panel, quantitation of CD68-positive stained area as a percentage of lesion area. **C-G**. Fluorescently labeled peritoneal exudate cells were injected intravenously into mice that had been treated with vehicle or PF-06282999 as described above. **C**. Protocol for leukocyte homing experiment. **D-E**. Aortas were extracted, and red fluorescent cells were counted in Z stacks using high content imaging. **D**. Left panel, representative aorta image demonstrating red fluorescent cells throughout the aorta, concentrating in the aortic arch. Right panel, close up of image in the white box. **E**. Quantitation of red cells per aorta normalized to aorta area as measured after pinning *en face*. Left panel, after 7 weeks of Western diet. Right panel, after 14 weeks of Western diet. **F-G**. Aortic roots were sectioned and stained for RFP and DAPI (images were taken at 5x magnification). **F**. Left panel, representative image of aortic root containing red fluorescent cells in the atherosclerotic lesion. Right panel, close up of image in white box. White arrowhead denotes RFP+ cell in aortic plaque. **G**. Quantitation of RFP positive stained area as a percentage of lesion area (N = 6–8, **; p<0.01). Aortic root images were taken at 5x magnification.

**Fig 4:**
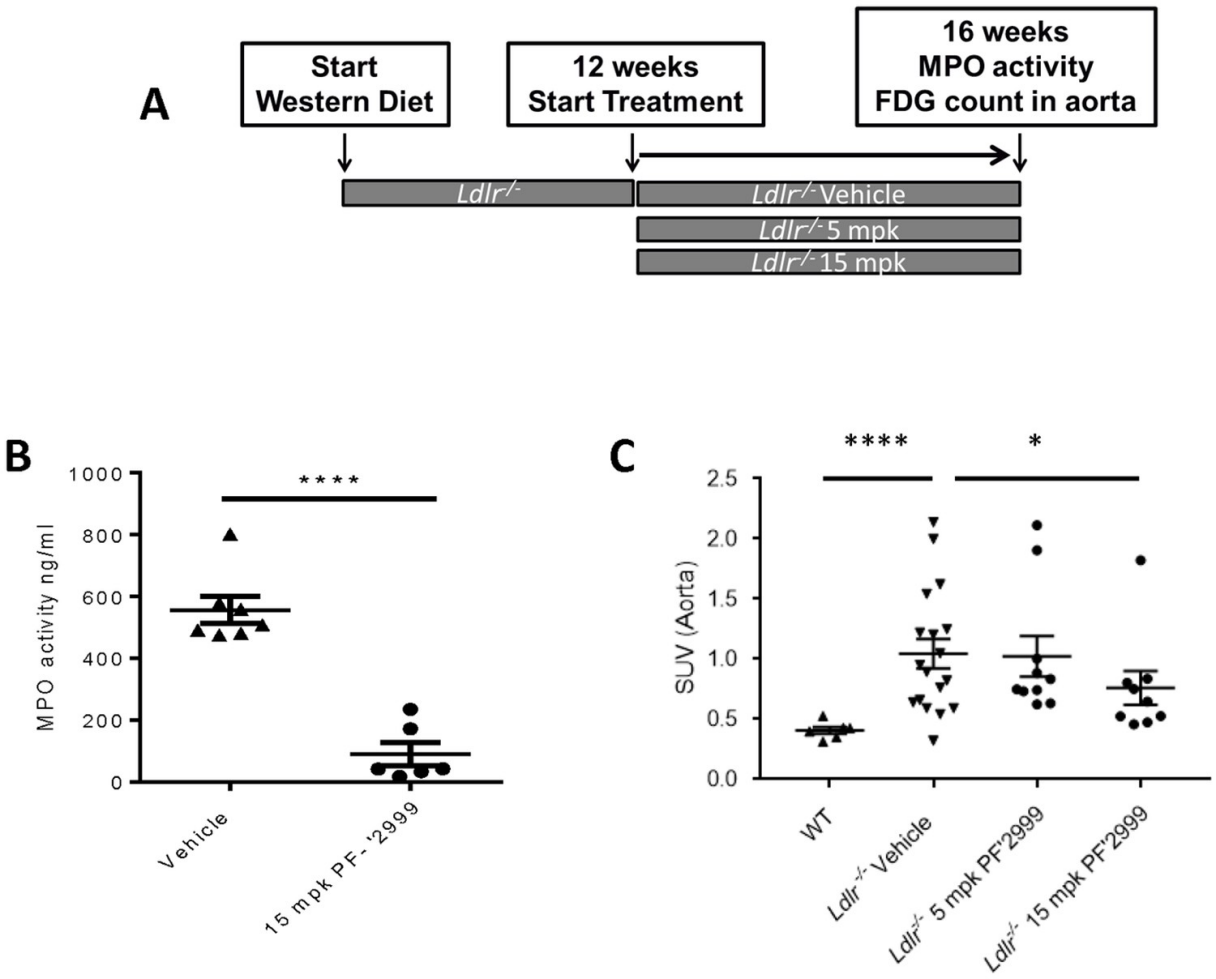
Reduced MPO activity and ^18^F-FDG in aortas after 4 weeks of MPO inhibitor treatment. *Ldlr*^-/-^ mice were fed Western diet for 12 weeks, at which time, treatment with 5 or 15 mg/kg MPO inhibitor PF-06282999 or vehicle PO, BID was performed for an additional 4 weeks. **A**. Study design. **B**. Aortas were extracted at the end of the study, and aortic MPO activity was measured one hour post dose. **C**. Animals were injected with 200–300μCi, <100μL ^18^F-FDG for 60 minutes, and aortas were extracted and counted (N = 5–18, **; p<0.01, ****; p<0.0001).

A two-tailed, two-sample Student’s t-test was performed in GraphPad Prism 7.04 (USA) for Figs 1, 2, 3B and 4B, and the WT-vehicle comparison in 4C. A p-value < 0.05 (or, *P* < 0.05) was used to determine significance. Fig 3D and 3F and a subset of 4C were analyzed for a dose response using a one-sided Jonckheere-Terpstra test for ordered alternatives in Cytel Studio 10.0 (USA).

## Results

### MPO activity is reduced yet lesion area is unaltered in *Ldlr*^-/-^ mice treated with MPO inhibitor PF-06282999

Myeloperoxidase is strongly linked with cardiovascular outcomes and atherosclerotic disease[30]. To assess mechanisms underlying MPO-mediated disease progression, we utilized the *Ldlr*^-/-^ mouse, which is a well characterized model of hyperlipidemia and atherosclerosis. *Ldlr*^-/-^ animals were fed Western diet for 16 weeks, and MPO inhibitor PF-06282999, a recently described small molecule MPO inhibitor[25], was administered throughout the entire study duration. Previous data described the IC_50_ of PF-06282999 inhibition of LPS-stimulated MPO activity in human whole blood to be 1900 nM, which corresponds to an unbound concentration of 724 nM[25]. PO administration of PF-06282999 (15 mg/kg) BID led to an average unbound concentration of 19382 ± 4264 nM (n = 7) 1 hour post dose. At study completion, plasma MPO activity was assessed; at the 15 mg/kg dose, MPO activity and total MPO levels were reduced by 85% and 44%, respectively (Fig 1A and 1B), which equated to an 73% reduction in MPO activity when normalized to MPO levels (Fig 1C).

Aortas were excised from mice treated with vehicle or PF-06282999, were prepared *en face*, and stained with Oil red O. Average aortic lesion area was quantified as the percentage of Oil red O stained area normalized to total aorta area. Interestingly, though MPO has been reported to drive atherosclerosis progression[31,32], lesion area did not differ between the two groups (10.5% vs. 13.9% lesion area for vehicle or PF-06282999 treated animals, respectively) (Fig 1C and 1D). Importantly, no significant change was observed in body weights of the animals throughout the study (not shown), and total cholesterol levels between the two groups were unaltered (2106 ± 89.74 vs. 2157 ± 75.07 mg/dL for vehicle vs. PF-06282999 treated, respectively), though oxidized cholesterol fractions were not assessed.

### Reduced necrotic core area after PF-06282999 treatment

Aortic roots were isolated from these animals and were sectioned and stained with hematoxylin and eosin. Total atherosclerotic lesion area was quantified, and no significant change was observed between animals treated with vehicle and those treated with PF-06282999 (Fig 2A and 2B). However, when necrotic core area was quantified, a significant 37% reduction in necrotic core area was observed in the atherosclerotic lesions from animals that had been treated with PF-06282999 compared with animals that were vehicle treated, which was also significant when normalized to total lesion area (Fig 2C). Masson’s Trichrome staining was then performed on the aortic root sections, and positive area was quantified as a measure of collagen content. Interestingly, there was a 25% increase in overall collagen content in animals that had been treated with PF-06282999 compared with those that had been vehicle treated (p = 0.07), which equated to a significant 77% increase in percent collagen area when normalized to lesion area (Fig 2D and 2E). Oil Red O staining was also performed, and no differences in overall Oil Red O stained area nor the percent of Oil Red O-positive area were observed (Fig 2F and 2G). Thus, MPO inhibition with PF-06282999 did not alter plaque area, but it did have a significant effect to reduce the development of the necrotic core in atherosclerotic plaques at the aortic root.

### Minor contribution of macrophage dynamics in mice treated with PF-06282999

In mice, atherosclerotic lesion size is driven primarily by recruitment of monocytes to sites of disturbed flow in the developing lesion followed by a proliferative response within the lesion[33]. Though MPO initiates and promotes inflammatory cascades[12], total lesion area in mice treated with MPO inhibitor PF-06282999 (Figs 1 and 2) was unaltered. Therefore, we assessed whether macrophage content was altered in MPO inhibitor-treated animals. In aortic root sections derived from the mice described in Figs 1 and 2, no changes in CD68 staining were observed with PF-06282999 compared with vehicle treatment (Fig 3A and 3B), suggesting that total macrophage content was unaltered. However, to rule out the possibility that MPO inhibition was affecting immune cell recruitment to atherosclerotic lesions, an immune cell homing experiment was performed as described in Fig 3C. Thioglycollate-elicited peritoneal exudate cells (PECs) were labelled with phagocytosed fluorescent microspheres and injected intravenously into animals that had been treated with vehicle or PF-06282999 (5 and 15 mg/kg) for 7 or 14 weeks. The mean unbound plasma exposure 1 hour post final PF-06282999 dose was 5301 ± 1846 nM (5 mg/kg) and 15958 ± 6327.9 nM (15 mg/kg). Animals were euthanized 48 hours after fluorescent PEC injection, and aortic roots as well as full aortas were harvested. Aortas were fixed, and fluorescent cells were imaged and quantified using high content imaging (Fig 3D and 3E).

At the 7 week time point, atherosclerotic lesion development in mice is driven predominantly by macrophage homing to the plaque[33], and at this time point we observed a dose-dependent significant reduction in fluorescent leukocytes within the aorta using PF-06282999 (Fig 3E). However, at the 14 week time point, much of atherosclerotic plaque size is driven by lesional macrophage proliferation[33]. Though an overall increase in fluorescent PECs was observed in the aortas compared with the 7 week time point (Fig 3E), when normalized to plaque area as measured in *en face* pinned aortas, the ratio of recruited PECs to plaque area was actually lower at 14 weeks compared with 7 weeks, which is consistent with the notion that plaque growth in this late stage is driven by macrophage proliferation (not shown). At the 14 week time point, no changes in plaque area were observed among the treatment groups (not shown), and the changes in PEC homing observed with MPO inhibitor treatment that had been observed at the 7 week time point were no longer observed (Fig 3E). Similarly, aortic roots were harvested after 14 weeks of dosing, and histological sections were prepared and imaged with fluorescence microscopy for the fluorescent microspheres and DAPI (Fig 3F). Fluorescent PECs were quantified as a percentage of total lesion area, and no changes were observed with MPO inhibitor treatment as compared with vehicle (Fig 3G). Fluorescent PECs were also quantified in the liver and spleen, and although a significant amount of fluorescent cells were observed in both tissues, MPO inhibition did not alter these values (not shown).

### Reduced MPO activity and ^18^F-FDG uptake in animals treated with MPO inhibitors

Because PF-06282999 treatment reduced necrotic core area, we asked whether it would also reduce biomarkers of plaque inflammation that may be clinically measurable. FDG-PET is an imaging modality that is commonly used to assess inflammation, and in atherosclerotic patients, alterations in carotid plaque FDG-PET signal are measurable and monitorable over time[34]. Furthermore, anti-inflammatory mechanisms such as anti-hypertensive therapies and statins may reduce FDG-PET signal in carotid plaques of human patients[34]. FDG-PET has also been used to assess plaque burden and inflammation in the *Apoe*^-/-^ model of atherosclerosis[35]. Thus, we sought to measure this parameter in the *Ldlr*^-/-^ model after MPO treatment. As a control experiment, we assessed whether MPO would be inhibited in established atherosclerotic plaques on the time scale of a therapeutic treatment regimen. Thus, *Ldlr*^-/-^ mice were fed Western diet for 12 weeks, at which time, animals were split into vehicle and 15 mg/kg PF-06282999 treatment groups and dosed BID for 4 weeks (Fig 4A). At euthanasia, aortic arches were dissected and lysed, and a significant 85% reduction in MPO activity was observed in mice that had been treated with 15 mg/kg PF-06282999 (Fig 4B).

We then sought to address whether MPO inhibition with PF-06282999 would reduce ^18^F-FDG signal in atherosclerotic mice. Due to the technical difficulty of imaging mouse aortas because of the small area and high background of heart and brown adipose tissue, we isolated aortas and other tissues subsequent to ^18^F-FDG injection and subjected them to scintillation counting. The ^18^F-FDG- signal was quantified as a measure of standard uptake value (SUV). As expected, a significant increase in ^18^F-FDG signal was observed in *Ldlr*^-/-^ mice that had been fed Western diet for 16 weeks, which have atherosclerotic plaques, compared with wild type C57Bl6/J control animals, which do not have plaques (Fig 4C). Interestingly, a dose responsive reduction of measured SUV was observed; though the sub-therapeutic dose of MPO inhibitor (5 mg/kg) did not have any effect on the ^18^F-FDG signal, animals that had been treated with the therapeutic treatment regimen of 15 mg/kg PF-06282999 for 4 weeks did demonstrate a significant reduction in ^18^F-FDG signal as compared with vehicle-treated atherosclerotic animals (Fig 4C). These results suggest that FDG-PET could be used as a biomarker to visualize the inhibition of MPO-mediated remodeling in atherosclerotic lesions in humans.

## Discussion

Increased MPO protein levels are associated with poor clinical outcomes in humans[21,30,36]. A large body of evidence indicates that MPO activity can promote cellular dysfunction by generation of reactive oxygen species including its enzymatic product HOCl, direct oxidation, or NO consumption[12,13,18]. Cardiovascular health and outcomes are tightly linked to cholesterol levels and atherosclerosis development, and MPO can promote lipoprotein oxidation[14,15,37,38]. Mouse models of atherosclerosis lacking MPO have provided controversial results, as these mice actually display increased plaque area[23]. However, MPO transgenic animals also display increased plaque content[31,32]. Thus, we sought here to assess whether inhibition of MPO activity with a highly specific MPO inhibitor would impact atherosclerotic plaque development in mice, and whether we would be able to monitor MPO-induced plaque remodeling in the intact animal. We observed that MPO inhibition with PF-06282999 at therapeutically relevant doses inhibited MPO activity and levels in the plasma of *Ldlr*^-/-^ mice but did not ameliorate atherosclerotic plaque formation in the aorta or the aortic root (Figs 1 and 2). Notably, even though chronic MPO inhibition with PF-06282999 reduced MPO levels in mice, the ratio of MPO activity to MPO levels was still reduced. However, even in the absence of alterations in atherosclerotic lesion size, mice treated with MPO inhibitor PF-06282999 displayed reduced necrotic core and increased collagen area as measured in aortic root histological sections (Fig 2). These data are consistent with the notion that MPO can activate matrix metalloproteinases, leading to collagen degradation and smooth muscle cell apoptosis[39,40]. These phenomena are associated with increased plaque vulnerability and rupture, which is a leading cause of cardiovascular death[20,40]. Previous studies with other MPO inhibitors have also demonstrated reductions in necrotic core area along with increased collagen content in mouse models of atherosclerosis and plaque vulnerability [8,41–43]. In addition to pharmacological MPO inhibitors, a number of additional strategies to diminish MPO activity such as thiocyanate or nitroxide radical supplementation have also provided similar benefits [43,44]. A better mechanistic understanding of how MPO inhibition alters inflammation, extracellular matrix composition, chlorination byproducts, and cellular death in atherosclerotic lesions will evaluated in future studies.

Interestingly, despite reductions in necrotic core area, changes in macrophage content in the aortic root lesions were not observed (Fig 3), and although there was reduced leukocyte homing to the aorta in early atherosclerotic lesions, MPO inhibition did not alter leukocyte homing to aorta or aortic root plaques in more advanced lesions (Fig 3). Because MPO is clearly involved in the initiation of inflammatory cascades in acute inflammatory processes[12], it is interesting that MPO inhibition did not alter macrophage content or leukocyte homing. Though we did not assess macrophage subtype in this study, M2 macrophages are associated with more stable atherosclerotic plaques [45], so a switch from a pro-inflammatory M1 subtype to an M2 subtype macrophage could have contributed to the beneficial effect that was observed on plaque remodeling. Further studies will be performed to assess these parameters. Finally, it has been demonstrated that lesional macrophage proliferation is the dominant mode of atherosclerotic lesion growth in advanced lesions, whereas recruitment is dominant in early lesions[33]. Therefore, we may have observed more pronounced differences in macrophage content if early atherosclerotic lesions had been assessed.

In addition to its role in atherosclerosis, MPO also drives the inflammatory process that occurs after cardiac injury, such as in myocardial infarct[29]. To this end, animals lacking MPO are also protected from cardiac dysfunction[24], and we have previously demonstrated in a mouse myocardial infarct model that inhibiting MPO activation with a similar MPO inhibitor ameliorated cardiac inflammation and scar formation, thus improving cardiac function[29]. The improvement in cardiac function was greater if MPO inhibitors were administered earlier in the course of the disease model, which correlated with reduced levels of neutrophils as well as CD11b positive, Ly6cHi macrophages; these results are consistent with the function of MPO to promote inflammatory cascades[19]. Taken together, these results suggest a benefit of MPO inhibition to remodel atherosclerotic plaques in addition to preventing cardiac inflammation after MI, which should have added benefit to prevent future cardiac dysfunction, as unstable plaques and previous myocardial infarct can promote future myocardial infarcts. Indeed, plasma MPO levels are prognostic for secondary cardiac events[22,30,36].

One challenge with developing MPO inhibitors is monitoring clinical activity at the site of action. Because the products of MPO activity are not completely specific to MPO, are in low abundance, and have very short half-lives, measuring MPO activity at the site of action is challenging. Recently, specific imaging agents have been developed to monitor MPO activity[29,46,47]. However, these agents are not yet available for clinical use. Thus, there is a need to develop methods to measure surrogate markers of MPO activity that correlate with disease outcomes. FDG PET is a commonly used agent that is taken up by inflamed carotid plaques in humans and can be modulated with anti-inflammatory therapies such as anti-hypertensive agents and statins[34]. Therefore, we asked whether ^18^F-FDG signal could be modulated by MPO inhibition using a therapeutic treatment regimen. We observed that 4 weeks of MPO inhibitor treatment was sufficient to reduce MPO activity within the mouse aortic arch, which contained atherosclerotic lesions (Fig 4). Furthermore, a dose dependent effect was observed in aortic ^18^F-FDG signal, as treatment of mice with a therapeutic dose, but not a sub-therapeutic dose of MPO inhibitor reduced this signal, suggesting that MPO inhibition in established plaques may induce remodeling. These data are consistent with human data demonstrating MPO expression in vulnerable plaques [10].

In summary, MPO inhibition did not affect atherosclerotic lesion size but did promote atherosclerotic plaque remodeling in mice, which is consistent with its known role in humans to promote cardiovascular disease. Furthermore, modulation of atherosclerotic plaque phenotype after MPO inhibitor treatment was observed by ^18^F-FDG, a widely used PET imaging agent that is modulated in correlation with plaque inflammation. These data suggest that MPO inhibition may have dual effects clinically to both improve myocardial function and reduce the likelihood of plaque rupture in vivo. Furthermore, the effects of MPO inhibitor treatment in plaques may be a clinically monitorable endpoint to predict patient response to treatment.

## Supporting information

S1 File Raw data supporting the means and standard error of the means that were used to generate all graphical data contained within the manuscript. (XLSX)
